# Anthropometry-adjusted TyG indices improve insulin resistance estimation: an exploratory euglycemic–hyperinsulinemic clamp study in Japanese adults without diabetes

**DOI:** 10.1007/s13340-026-00883-9

**Published:** 2026-04-05

**Authors:** Natsu Otowa-Suematsu, Tomoaki Nakamura, Hiroshi Miura, Tomoko Yamada, Marika Nishisaka, Hayato Fukumitsu, Yukari Katsura, Yasuko Morita, Shun-Ichiro Asahara, Kazuhiko Sakaguchi

**Affiliations:** 1 Division of Diabetes and Endocrinology, Department of Internal Medicine, Kobe University Graduate School of Medicine, Kobe, 650-0017 Japan; 2 Department of Diabetes and Endocrinology, Kakogawa Central City Hospital, Kakogawa, 675-8611 Japan; 3 Department of Diabetes and Endocrinology, Takatsuki General Hospital, Takatsuki, 569-1192 Japan; 4 Division of Community Medicine and Medical Education, Department of Social/Community Medicine and Health Science, Kobe University Graduate School of Medicine, Kobe, 650-0017 Japan

**Keywords:** Triglyceride-glucose index, Anthropometric indices, Insulin resistance, Glucose clamp

## Abstract

**Background:**

We evaluated the diagnostic performance of anthropometry-adjusted triglyceride-glucose (TyG)-derived indices for assessing insulin resistance (IR) in Japanese adults without diabetes, using the euglycemic–hyperinsulinemic clamp (EHC) as the reference standard.

**Methods:**

A total of 61 Japanese individuals without diabetes underwent insulin sensitivity assessment using the gold standard EHC. IR was defined as an insulin sensitivity index (ISI) below the 25th percentile. Surrogate IR indices, including Homeostasis Model Assessment of Insulin Resistance (HOMA-IR), the original TyG index, and anthropometry-adjusted TyG-derived indices (TyG-BMI, TyG-WC, TyG-WHtR), were calculated. Correlations between these indices and the ISI were evaluated using Spearman’s rank correlation. Receiver operating characteristic (ROC) analysis and linear regression were used to compare diagnostic accuracy and predictive ability. Pairwise DeLong tests were used to assess differences in the area under the curve (AUC) values.

**Results:**

TyG-BMI and TyG-WHtR showed the stronger associations with ISI (ρ = − 0.544 and − 0.546, respectively) than both HOMA-IR and the original TyG index. TyG-BMI and TyG-WHtR had the highest AUCs (0.810 and 0.829, respectively) for identifying clamp-defined IR. The optimal cutoff value for HOMA-IR was approximately 2.5. The optimal cutoff for TyG-BMI was 203.7. Pairwise DeLong tests supported the higher diagnostic performance of selected anthropometry-adjusted TyG-derived indices compared with conventional markers.

**Conclusions:**

Anthropometry-adjusted TyG-derived indices, particularly TyG-BMI and TyG-WHtR, showed strong associations with IR as assessed by the gold standard EHC in Japanese adults without diabetes. These findings suggest that these indices may serve as practical surrogate markers for IR in settings where direct clamp measurements are not feasible.

**Supplementary Information:**

The online version contains supplementary material available at 10.1007/s13340-026-00883-9.

## Introduction

Insulin resistance (IR) is a fundamental pathophysiological condition underlying a variety of diseases and disorders, including diabetes mellitus [1], hypertension [2], metabolic dysfunction-associated steatotic liver disease (MASLD) [3], cardiovascular diseases [4], and malignancies [5]. Early evaluation of IR is crucial for the prevention and timely intervention of these conditions. The gold standard for assessing IR is the euglycemic-hyperinsulinemic clamp (EHC), as originally described by DeFronzo [6]. However, the EHC requires specialized equipment, skilled personnel, and substantial time and cost, making its widespread application in clinical practice challenging. Therefore, several surrogate markers have been developed for the simplified evaluation of IR and are frequently used in clinical settings.

Among these, the Homeostasis Model Assessment of Insulin Resistance (HOMA-IR) is the most widely used index, due to its simplicity compared with the EHC [7]. Elevated HOMA-IR values are reportedly associated with various clinical outcomes, including the development of diabetes [8]. However, the calculation of HOMA-IR requires both fasting plasma glucose and fasting insulin levels, which limits its use in patients receiving insulin therapy and makes it susceptible to analytical issues such as hemolysis. Furthermore, fasting insulin is not routinely measured in daily clinical practice or health checkups, thereby restricting the practical applicability of HOMA-IR in large-scale studies and real-world clinical settings.

The Matsuda index is another indicator for IR evaluation and has shown a strong correlation with the glucose infusion rate (GIR) obtained from the EHC [9]. Nevertheless, this index requires a time-consuming 75-g oral glucose tolerance test (OGTT) and relies on insulin measurements, making it less suitable for large population-based studies. Thus, there remains a need for simpler and more accurate markers for diagnosing IR.

Against this background, the triglyceride-glucose (TyG) index—an IR marker calculated from fasting plasma glucose and fasting triglyceride levels—has been proposed [10]. The TyG index does not require insulin measurements and is inexpensive, allowing its use in large-scale studies. Associations with various adverse clinical outcomes, including cardiovascular disease, liver dysfunction, and chronic kidney disease (CKD), have also been reported [11]. Furthermore, the TyG index has been reported to show better predictive performance than HOMA-IR in predicting metabolic syndrome and other IR-related conditions in diverse populations [12].

Recently, several TyG-derived indices adjusted for anthropometric parameters, such as body mass index (TyG-BMI), waist circumference (TyG-WC), and waist-to-height ratio (TyG-WHtR), have been developed and have demonstrated strong associations with clinical outcomes [13–17]. Notably, TyG-derived indices have stronger associations with clinical outcomes than the original TyG index [18, 19]. Although evidence supporting the utility of TyG-derived indices is accumulating, no study has directly compared these indices with the gold standard EHC for IR assessment.

Through this study, we aimed to evaluate the diagnostic performance of TyG-derived indices for IR in Japanese individuals without diabetes, using the EHC as the reference standard. By directly comparing these indices against the reference standard, this study provides evidence that they can serve as simple, cost-effective alternatives to clamp-based assessments, with implications for clinical practice and large-scale epidemiological research.

## Materials and methods

### Study design and participants

This study was approved by the Ethics Committee of Kobe University Hospital (Approval No. 1834) and conducted in accordance with the Declaration of Helsinki and its amendments. Written informed consent was obtained from all participants prior to enrollment.

Adults aged 20 years or older without a prior diagnosis of diabetes were recruited at Kobe University Hospital (Hyogo, Japan) between January 2016 and March 2018. Participants were excluded if they were receiving medications known to affect glucose metabolism (such as steroids or beta-blockers), had psychiatric disorders, were pregnant, or lactating. Each participant underwent a standard 75-g OGTT in the morning after an overnight fast. Participants underwent EHC tests using an artificial endocrine pancreas (STG-55; Nikkiso Co., Tokyo, Japan) within 7 days after the OGTT.

### OGTT procedures

A standard 75-g OGTT was performed after an overnight fast. Venous blood samples were collected at 0, 30, 60, 90, and 120 min for measurements of plasma glucose and serum insulin concentrations. Participants who met the World Health Organization diagnostic criteria for diabetes were excluded from the present analysis.

### EHC procedures

Within 7 days after the OGTT, insulin sensitivity was assessed using the gold standard EHC technique with an artificial endocrine pancreas system (STG-55; Nikkiso, Tokyo, Japan) according to previously established methods [20, 21]. Teflon catheters were placed in the antecubital veins of both arms: one for blood sampling and the other for the infusion of glucose and insulin. For glucose measurement, blood samples were obtained from the sampling catheter while the arm was locally warmed with disposable chemical heat packs to obtain arterialized venous blood. In brief, a continuous infusion of human regular insulin was administered at a rate of 40 mU m⁻^2^ min⁻^1^, and plasma glucose was maintained at approximately 90 mg/dL by variable glucose infusion.

### Analytical sample

A total of 70 individuals were initially enrolled. After exclusion of participants with protocol deviations, missing data, or medications affecting glucose metabolism, 64 were eligible. For the present analysis, we excluded three individuals newly diagnosed with diabetes by OGTT, yielding a final sample of 61 Japanese adults without diabetes.

### Calculation of IR indices

IR was assessed using several validated indices. All surrogate indices of IR were calculated using measurements obtained on the OGTT day, whereas insulin sensitivity index (ISI) was derived from the clamp procedure.

The HOMA-IR was calculated from fasting values as follows:

$$ \begin{aligned} {\mathrm{HOMA}} - {\mathrm{IR}} = & \left[ {{\mathrm{fasting}}\,{\mathrm{insulin}}\,\left( {\mu {\mathrm{U}}/{\mathrm{mL}}} \right)} \right. \\ & \left. { \times {\mathrm{fasting}}\,{\mathrm{plasma}}\,{\mathrm{glucose}}\,\left( {{\mathrm{mg}}/{\mathrm{dL}}} \right)} \right]/405 \\ \end{aligned} $$

The triglyceride-glucose index (TyG index) was calculated as follows:

$$\begin{aligned} {\mathrm{TyG}}\,{\mathrm{index}} = & {\text{ln }}\left[ {{\mathrm{fasting}}\,{\mathrm{triglycerides}}\,\left( {{\mathrm{mg}}/{\mathrm{dL}}} \right)} \right. \\ & \left. { \times {\mathrm{fasting}}\,{\mathrm{glucose}}\,\left( {{\mathrm{mg}}/{\mathrm{dL}}} \right)} /2 \right]\\ \end{aligned} $$

TyG-derived indices were calculated by multiplying the TyG index by anthropometric measures of body size or fat distribution as follows:

$$ {\mathrm{TyG}} - {\mathrm{BMI}} = {\mathrm{TyG}}\,{\mathrm{index}} \times {\mathrm{BMI}}\,\left( {{\mathrm{kg}}/{\mathrm{m}}^{{\mathrm{2}}} } \right) $$

$$ {\mathrm{TyG}} - {\mathrm{WC}} = {\mathrm{TyG}}\,{\mathrm{index}} \times {\mathrm{waist}}\,{\mathrm{circumference}}\left( {{\mathrm{cm}}} \right) $$

$$ {\mathrm{TyG}} - {\mathrm{WHtR}} = {\mathrm{TyG}}\,{\mathrm{index}} \times \left( {{\mathrm{waist}}\,{\mathrm{circumference}}/{\mathrm{height}}} \right) $$

The Matsuda index was calculated from glucose and insulin values obtained during the OGTT, as previously described [9]:

$$ \begin{aligned} & {\mathrm{Matsuda}}\,{\mathrm{index}} \\ & \, = 10,000/\surd \left[ {{\mathrm{fasting}}\,{\mathrm{glucose}} \times {\mathrm{fasting}}\,{\mathrm{insulin}} \times {\mathrm{mean}}\,{\mathrm{glucose}} \times {\mathrm{mean}}\,{\mathrm{insulin}}} \right] \\ \end{aligned} $$

where mean glucose and mean insulin represent the arithmetic means of the respective values at 0, 30, 60, 90, and 120 min.

The ISI was calculated as previously described [22], using the mean GIR during the final 30 min of the 120-min EHC, divided by the product of plasma glucose and serum insulin levels at the end of the clamp, multiplied by 100.

$$\begin{aligned} {\mathrm{ISI}} = & \left[ {{\mathrm{mean}}\,{\mathrm{GIR}}/\left( {{\mathrm{plasma}}\,{\mathrm{glucose}}} \right.} \right. \\ & \left. {\left. { \times {\mathrm{serum}}\,{\mathrm{insulin}}\,{\mathrm{at}}\,{\mathrm{the}}\,{\mathrm{end}}\,{\mathrm{of}}\,{\mathrm{the}}\,{\mathrm{clamp}}} \right) }\right] \times 100\\ \end{aligned} $$

### Statistical analysis

The distribution of each continuous variable was assessed using the Shapiro–Wilk test. Normally distributed variables were expressed as mean ± standard deviation (SD), whereas non-normally distributed variables were presented as median and interquartile range (IQR). In this study, IR was defined as the lowest quartile of the ISI derived from the EHC, because no universally accepted absolute cutoff for clamp-derived insulin sensitivity has been established in Japanese populations. This definition was intended as a cohort-specific classification for internal comparison rather than a clinically or externally validated threshold.

Comparisons between the IR (+) and IR (−) groups were performed using either the independent samples *t*-test or the Mann–Whitney *U* test, depending on the normality of the data.

Spearman’s rank correlation coefficients were calculated for HOMA-IR, Matsuda index, TyG index, TyG-BMI, TyG-WC, and TyG-WHtR to assess associations between ISI and surrogate indices of IR. Additionally, simple linear regression analysis was conducted to evaluate each surrogate index’s ability to predict ISI as a continuous variable. Coefficients of determination (*R*^*2*^) and residual sums of squares were calculated to assess the fit of each model.

Receiver operating characteristic (ROC) curve analyses were also conducted to evaluate each index’s ability to identify IR based on the ISI threshold. Given that the Matsuda index is inversely associated with IR, ROC analyses were conducted by specifying the appropriate direction of test positivity without transforming the original values. For each ROC analysis, the area under the curve (AUC), *p*-values (testing whether each AUC differs significantly from 0.5), optimal cutoff points, and Youden index values were obtained. To assess the robustness of the ROC analyses given the limited sample size, we performed a bootstrap-based sensitivity analysis with 10,000 resamples to estimate the stability and 95% confidence intervals(Bootstrap 95% CI) of AUC values for each surrogate index. Pairwise comparisons of AUCs were performed using the DeLong test, focusing on comparisons between the two primary anthropometry-adjusted TyG-derived indices (TyG-BMI and TyG-WHtR) and conventional surrogate markers (HOMA-IR and the original TyG index). To control for multiplicity arising from these focused comparisons (*m* = 4), *p*-values were adjusted using the Holm method. Additional regression analyses were performed to evaluate the incremental contribution of anthropometric measures to the TyG index. IR (defined as the lowest quartile of clamp-derived ISI) was analyzed using logistic regression with additive (TyG + anthropometry) and interaction (TyG × anthropometry) models, which were compared using likelihood ratio tests. Model performance was assessed by AUC with bootstrap-based optimism correction.

All statistical analyses were performed using SPSS Statistics version 29 (IBM Corp., Armonk, NY, USA). Pairwise comparisons of AUCs between the novel TyG-derived indices and traditional markers (HOMA-IR, TyG index) were conducted using the DeLong test in R version 4.3.2 (R Foundation for Statistical Computing, Vienna, Austria). Bootstrap-based estimation of AUC stability and 95% confidence intervals was performed using Python (version 3.8.8; Python Software Foundation). Additive and interaction models were both implemented in Python. All tests were two-sided, and *p* < 0.05 was considered statistically significant.

## Results

### Study participants

Participant characteristics are shown in Table 1. A total of 61 individuals were included in the analysis (median age: 34.0 years; mean BMI: 22.5 kg/m^2^). The cohort comprised 52 individuals with normal glucose tolerance and 9 with impaired glucose tolerance.

**Table 1:** Clinical characteristics of study participants

Characteristic	All (n = 61)	IR(−) Group (n = 46)	IR(+) Group (n = 15)	*p*-value
*Clinical*				
Male (*n* [%])	45 [73.8]	33 [71.7]	12 [80.0]	0.738
Age (years)	34.0 [29.5 to 42.0]	33.0 [28.8 to 42.0]	38.0 [34.0 to 42.0]	0.142
Height (cm)	172.4 [160.9 to 177.3]	172.6 [160.6 to 177.1]	171.0 [161.2 to 178.0]	0.913
Body weight (kg)	65.4 ± 12.8	62.7 ± 11.8	73.4 ± 12.9	0.004
BMI (kg/m^2^)	22.5 ± 2.9	21.6 ± 2.2	25.2 ± 2.9	< 0.001
Waist circumference (cm)	79.4 ± 9.7	77.0 ± 9.0	86.8 ± 8.2	< 0.001
NGT / IGT (*n*)	52 / 9	43 / 3	9 / 6	0.005
*Laboratory data*				
Creatinine (mg/dL)	0.70 ± 0.14	0.68 ± 0.13	0.77 ± 0.13	0.034
eGFR (mL/min/1.73m^2^)	99.5 [83.7 to 113.3]	102.2 [90.1 to 113.7]	84.7 [79.5 to 99.5]	0.017
AST (U/L)	19.0 [17.0 to 23.0]	19.0 [17.0 to 22.0]	23.0 [19.0 to 26.0]	0.018
ALT (U/L)	16.0 [12.0 to 26.5]	14.0 [11.0 to 21.3]	26.0 [15.0 to 37.0]	0.005
TG (mg/dL)	71.0 [51.0 to 109.5]	67.0 [49.0 to 91.0]	105.0 [77.0 to 147.0]	0.007
LDL-C (mg/dL)	114.0 [94.0 to 126.5]	112.0 [92.0 to 121.0]	130.0 [97.0 to 166.0]	0.061
HDL-C (mg/dL)	55.0 [48.0 to 62.0]	56.5 [50.0 to 62.0]	52.0 [41.0 to 70.0]	0.169
Total cholesterol (mg/dL)	189.0 [166.0 to 202.5]	188.5 [166.5 to 198.3]	203.0 [165.0 to 241.0]	0.150
HbA1c (%)	5.13 ± 0.24	5.13 ± 0.24	5.14 ± 0.28	0.873
FPG (mg/dL)	91.3 ± 6.6	91.5 ± 6.8	90.7 ± 6.2	0.677
F-IRI (µIU/mL)	7.0 [5.25 to 10.05]	6.6 [5.2 to 8.7]	8.6 [6.8 to 17.9]	0.051
*Metabolic Indices*				
HOMA-IR	1.55 [1.21 to 2.16]	1.44 [1.17 to 1.94]	2.06 [1.51 to 3.88]	0.053
Matsuda index	5.97 [4.09 to 7.18]	6.18 [4.63 to 7.36]	3.62 [2.73 to 6.17]	0.004
TyG index	8.15 ± 0.55	8.04 ± 0.50	8.47 ± 0.59	0.007
TyG-BMI	179.5 [158.2 to 205.6]	175.3 [155.8 to 191.4]	212.3 [176.8 to 252.9]	< 0.001
TyG-WC	650.1 ± 112.0	621.1 ± 95.5	739.1 ± 114.8	< 0.001
TyG-WHtR	3.73 [3.36 to 4.16]	3.56 [3.33 to 3.93]	4.29 [3.78 to 4.68]	< 0.001
ISI (mg/kg/min per µU/mL per mg/dL)	0.116 [0.072 to 0.150]	0.126 [0.106 to 0.181]	0.050 [0.049 to 0.065]	< 0.001

Data are expressed as means ± SD or median (IQR) for continuous variables, and as number (percentage) for categorical variables

*P*-values indicate comparisons between IR(+) and IR(−) groups using the *t*-test or the Mann–Whitney *U* test for continuous variables, and the chi-squared test for categorical variables

IR, insulin resistance; BMI, body mass index; NGT, normal glucose tolerance; IGT, impaired glucose tolerance; eGFR, estimated glomerular filtration rate; AST, aspartate aminotransferase; ALT, alanine aminotransferase; TG, triglyceride; LDL-C, low-density lipoprotein cholesterol; HDL-C, high-density lipoprotein cholesterol; HbA1c, hemoglobin A1c; FPG, fasting plasma glucose concentration; F-IRI, fasting serum immunoreactive insulin concentration; HOMA-IR, homeostasis model assessment of insulin resistance; TyG index, triglyceride-glucose index; TyG-BMI, triglyceride-glucose body mass index; TyG-WC, triglyceride-glucose waist circumference; TyG-WHtR, triglyceride-glucose waist-to-height ratio; ISI, insulin sensitivity index

No significant differences were observed between the IR (−) and IR (+) groups in terms of age, sex distribution, HbA1c, or fasting plasma glucose level. However, BMI, waist circumference, and triglyceride (TG) levels were significantly higher in the IR (+) group (*p* < 0.001, *p* < 0.001, and *p* = 0.007, respectively). HOMA-IR tended to be higher in the IR (+) group (*p* = 0.053). The TyG index and all TyG-derived indices were significantly elevated in the IR (+) group (TyG index: *p* = 0.007; TyG-BMI: *p* < 0.001; TyG-WC: *p* < 0.001; TyG-WHtR: *p* < 0.001), whereas the Matsuda index was significantly lower (*p* = 0.004).

### Association between ISI and surrogate indices of IR

**Fig. 1:**
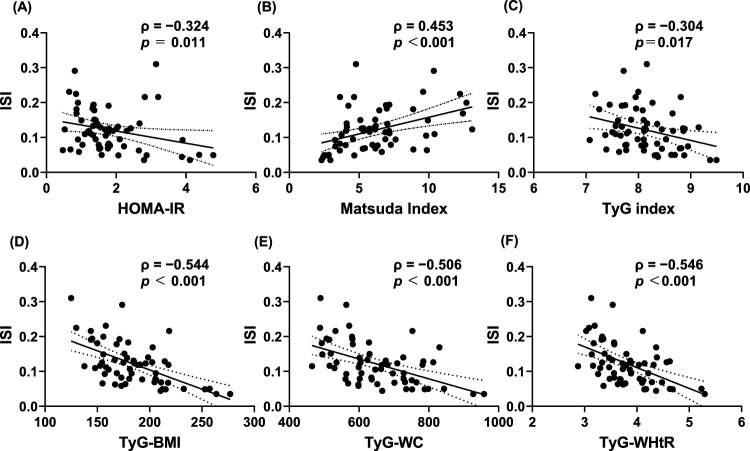
Correlation between insulin sensitivity index (ISI) and: **A** HOMA-IR, **B** Matsuda index, **C** TyG index, **D** TyG-BMI, **E** TyG-WC, and (**F**) TyG-WHtR. The solid line represents linear regression; dotted lines indicate the 95% confidence interval. Spearman’s correlation coefficient (ρ) and *p*-value are shown. HOMA-IR, homeostasis model assessment of insulin resistance; TyG index, triglyceride-glucose index; TyG-BMI, triglyceride-glucose body mass index; TyG-WC, triglyceride-glucose waist circumference; TyG-WHtR, triglyceride-glucose waist-to-height ratio

Figure 1 illustrates the correlations between the insulin sensitivity index (ISI) and various surrogate markers of IR. All indices showed significant correlations with ISI. Notably, TyG-derived indices (TyG-BMI, TyG-WC, and TyG-WHtR) demonstrated stronger correlations with ISI (ρ =  − 0.544, − 0.506, and − 0.546, respectively) compared to traditional indices, such as HOMA-IR and the TyG index (Fig. 1).

In sex-stratified subgroup analyses, TyG-derived indices (TyG-BMI, TyG-WC, and TyG-WHtR) showed inverse associations with ISI in both men and women, with similar directions of association across sexes. However, these subgroup analyses should be interpreted cautiously given the limited number of female participants. By contrast, associations for HOMA-IR, the original TyG index, and the Matsuda index were less consistent across sexes, and when present, the strength of the correlations was relatively modest (Supplementary Table 1).

Simple linear regression analysis of ISI demonstrated that the coefficients of determination (*R*^2^) for TyG-BMI and TyG-WHtR were higher, and the residual sums of squares were lower, than those for HOMA-IR and the TyG index (Table 2). Based on this model, the relationships between TyG-derived indices and ISI can be expressed as follows:

**Table 2:** Simple linear regression analysis between ISI and metabolic indices

Parameter	*R*^*2*^	Residual sum of squares
HOMA-IR	0.075	0.210
Matsuda index	0.179	0.186
TyG index	0.098	0.204
TyG-BMI	0.335	0.151
TyG-WC	0.270	0.166
TyG-WHtR	0.297	0.159

ISI, insulin sensitivity index;* R*^*2*^, coefficient of determination; HOMA-IR, homeostasis model assessment of insulin resistance; TyG index, triglyceride-glucose index; TyG-BMI, triglyceride-glucose body mass index; TyG-WC, triglyceride-glucose waist circumference; TyG-WHtR, triglyceride-glucose waist-to-height ratio

- For TyG-BMI:
  $$ \begin{aligned} & \mathrm{ISI} = 0.324 - 0.001 \times \left( {\mathrm{TyG} -\text{ BMI}} \right) \\ & \left( {R^{2} = 0.{335},\,p < 0.00{1}} \right) \\ \end{aligned} $$
- For TyG-WHtR:
  $$ \begin{gathered} \mathrm{ISI} = 0.35 - 0.06 \times \left( {\mathrm{TyG} - \mathrm{WHtR}} \right) \hfill \\ \left( {R^{2} = 0.{297},\,p < 0.00{1}} \right) \hfill \\ \end{gathered} $$

### Diagnostic performance of surrogate indices for ISI-defined IR

ROC curve analysis was conducted to evaluate the ability of various surrogate indices to identify IR, defined as the lowest quartile of the ISI derived from the EHC.

**Fig. 2:**
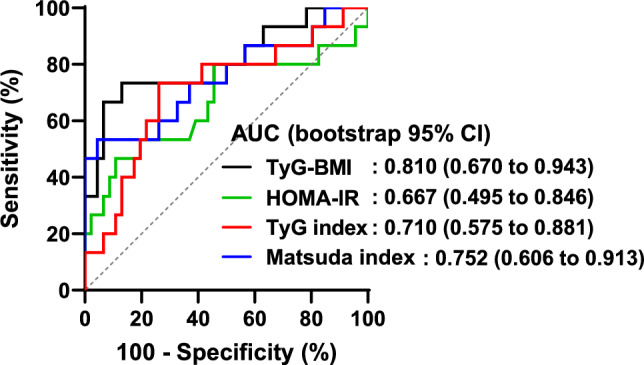
Receiver operator curves for the prediction of insulin sensitivity index (ISI). The black line indicates TyG-BMI, the green line indicates HOMA-IR, the red line indicates TyG index, and the blue line indicates Matsuda index. The dotted line represents the line of no discrimination, which corresponds to an AUC of 0.5. For the Matsuda index, ROC analyses were performed by specifying lower values as indicating insulin resistance. HOMA-IR, homeostasis model assessment of insulin resistance; TyG index, triglyceride-glucose index; TyG-BMI, triglyceride-glucose body mass index; AUC, area under the curve

Among the indices evaluated, TyG-BMI, TyG-WHtR, and TyG-WC demonstrated the highest discriminative performance, with AUCs of 0.810, 0.829, and 0.783, respectively (Table 3**, **Fig. 2). These AUC values were all significantly greater than 0.5, indicating meaningful discrimination (all* p* < 0.05). The anthropometry-adjusted TyG-derived indices showed better discriminative performance than traditional surrogate indices, such as HOMA-IR (AUC = 0.667, *p* = 0.053 vs. 0.5) and the TyG index (AUC = 0.710, *p* = 0.015 vs. 0.5). Although the Matsuda index exhibited moderate diagnostic ability (AUC = 0.752, *p* = 0.004 vs. 0.5), TyG-derived indices demonstrated higher sensitivity and greater overall discriminatory performance, as reflected by larger AUC and Youden index values.

**Table 3:** Diagnostic performance of metabolic indices for detecting insulin resistance

	AUC (apparent; Bootstrap 95% CI)	AUC (optimism-corrected; Bootstrap 95% CI)	*p*	Optimal cutoff (exploratory)	Sensitivity	Specificity	Youden index
HOMA-IR	0.667 (0.495 to 0.846)	0.682 (0.511 to 0.873)	0.053	2.54	0.467	0.891	0.358
Matsuda index	0.752 (0.606 to 0.913)	0.773 (0.630 to 0.939)	0.004	3.63	0.533	0.957	0.490
TyG index	0.710 (0.575 to 0.881)	0.738 (0.593 to 0.907)	0.015	8.29	0.733	0.739	0.472
TyG-BMI	0.810 (0.670 to 0.943)	0.820 (0.697 to 0.968)	< 0.001	203.74	0.733	0.870	0.603
TyG-WC	0.783 (0.657 to 0.913)	0.796 (0.678 to 0.934)	0.001	730.10	0.600	0.870	0.470
TyG-WHtR	0.829 (0.707 to 0.932)	0.833 (0.732 to 0.957)	< 0.001	4.08	0.733	0.804	0.537

P-values indicate the significance of the area under the curve for each index in detecting insulin resistance

Youden-derived cutoffs and threshold-dependent metrics are exploratory and cohort-specific

Abbreviation: AUC, area under the curve; HOMA-IR, homeostasis model assessment of insulin resistance; TyG index, triglyceride-glucose index; TyG-BMI, triglyceride-glucose body mass index; TyG-WC, triglyceride-glucose waist circumference; TyG-WHtR, triglyceride-glucose waist-to-height ratio

Among all indices, TyG-BMI yielded the highest Youden index (0.603), with a sensitivity of 73.3% and specificity of 87.0%, indicating the most favorable trade-off between true-positive and true-negative rates.

Pairwise comparisons using the DeLong test, with *p*-values adjusted for multiplicity using the Holm method (*m* = 4), supported these findings. The AUC of TyG-WHtR was significantly greater than that of the TyG index (adjusted *p* = 0.027). The other prespecified pairwise differences did not reach statistical significance after Holm adjustment, although the adjusted *p*-values were close to the threshold (TyG-BMI vs. TyG index, adjusted *p* = 0.055; TyG-WHtR vs. HOMA-IR, adjusted *p* = 0.057; TyG-BMI vs. HOMA-IR, adjusted *p* = 0.060).

Bootstrap analyses demonstrated that the AUC estimates for TyG-BMI and TyG-WHtR remained stable across resamples, supporting the robustness of their superior discriminative performance despite the limited sample size.

To evaluate the robustness of our finding against the definition of IR, we performed sensitivity analyses using alternative ISI thresholds. IR was redefined as the lowest 20% and the lowest 30% of ISI values, and ROC analyses were repeated for all surrogate indices. As shown in Supplementary Table 2, the overall ranking and relative diagnostic performance of the TyG-derived indices were largely consistent across these alternative definitions. In particular, TyG-BMI and TyG-WHtR continued to demonstrate comparatively higher AUC values than conventional indices. These results suggest that the superior discriminative ability of anthropometry-adjusted TyG-derived indices was not dependent on a single, arbitrary ISI threshold.

To further explore potential sex-specific differences, we conducted ROC analyses separately in men and women using the primary definition of IR (ISI in the lowest quartile). The sex-stratified AUC values and corresponding 95% CI are presented in Supplementary Table 3, and the corresponding ROC curves are shown in Supplementary Fig. 1. In both men and women, TyG-derived indices tended to show higher AUC values compared with HOMA-IR or the original TyG index.

### Incremental predictive value of anthropometric measures

Because clamp-derived indices can be influenced by body size and fat-free mass, we additionally evaluated anthropometric measures alone and formally tested whether combining TyG with anthropometry provides incremental information beyond the additive model. Detailed results of additive and interaction models, including optimism-corrected AUCs obtained by bootstrap resampling, are provided in Supplementary Table 4. Anthropometric measures alone demonstrated substantial discriminative performance for IR (AUCs: BMI 0.824; WC 0.779; WHtR 0.831), exceeding that of TyG alone (AUC 0.739). In logistic regression models, adding anthropometric measures to the TyG index significantly improved model fit. Specifically, the adding BMI to TyG significantly improved the model fit, as assessed by the likelihood ratio testing (*p* = 0.000553) and increased the AUC from 0.739 to 0.836. Similar improvements were observed when WC (*p* = 0.0213; AUC 0.796) or WHtR (p = 0.00122; AUC 0.840) was added to the TyG index. In contrast, inclusion of an interaction term (TyG × BMI, TyG × WC, TyG × WHtR) did not improve fit (all *p* > 0.17).

## Discussion

In this study, we demonstrated that anthropometry-adjusted TyG-derived indices, particularly TyG-BMI and TyG-WHtR, exhibited strong correlations with ISI as assessed by the gold-standard EHC in individuals without diabetes in Japan. These indices showed better performance than conventional surrogate markers, such as HOMA-IR and the original TyG index, in correlation strength and diagnostic performance, representing a key finding of this study.

Previous studies have evaluated the relationship between the conventional TyG index and IR assessed by EHC in various populations, including a Mexican cohort with heterogeneous glucose tolerance [23] and Chinese patients with type 2 diabetes [24]. These studies consistently demonstrated that the TyG index strongly correlates with IR determined by the EHC, regardless of sex, obesity status, or glucose tolerance. This finding supports its clinical utility as a simple surrogate marker for IR. Additionally, a large cross-sectional study in Korean adults demonstrated that TyG-BMI, TyG-WC, and TyG-WHtR had superior discriminative power for detecting IR defined by HOMA-IR compared with the TyG index alone [25]. However, no previous study has directly assessed the utility of anthropometry-adjusted TyG-derived indices using EHC as the reference standard.

Given that the GIR obtained from the EHC is inherently adjusted for body weight, it is physiologically reasonable to use anthropometry-adjusted TyG-derived indices. The ISI primarily reflects peripheral insulin sensitivity, particularly skeletal muscle glucose uptake, as hepatic glucose production is almost completely suppressed under EHC conditions. Incorporating anthropometric measures, such as BMI or WHtR, allows the TyG index to more effectively capture peripheral IR, which is influenced by overall adiposity. In this study, TyG-BMI and TyG-WHtR showed the strongest correlations with the ISI, as well as superior AUCs, Youden indices, and coefficients of determination (*R*^*2*^), along with the lowest residual sum of squares compared with conventional markers. Furthermore, pairwise DeLong tests confirmed that the AUC of TyG-WHtR was significantly higher than those of HOMA-IR and the TyG index, while TyG-BMI also showed better performance than the TyG index. Although visceral fat is generally considered more closely associated with IR than overall adiposity, TyG-BMI showed a slightly stronger correlation with ISI than TyG-WC in the present study. Because ISI derived from the euglycemic–hyperinsulinemic clamp primarily reflects peripheral insulin sensitivity, BMI may better capture interindividual differences relevant to skeletal muscle glucose uptake, particularly in relatively lean populations. This observation does not negate the importance of visceral fat but suggests that BMI-adjusted TyG indices may serve as a pragmatic surrogate for peripheral IR. Taken together, these findings suggest that incorporating anthropometric adjustment enables the TyG index to reflect the physiological determinants of IR more accurately. Importantly, additional analyses demonstrated that the apparent superiority of TyG-BMI, TyG-WC, and TyG-WHtR is partly attributable to the strong contribution of anthropometric measures to clamp-based insulin resistance. Although combining TyG with anthropometric measures improved consistency with clamp-derived indices in an additive manner, we found no evidence of a multiplicative (synergistic) effect beyond additivity. Accordingly, these anthropometry-adjusted TyG-derived indices should be interpreted as pragmatic composite markers of insulin resistance rather than mechanistically distinct measures.

In this study, IR was defined as an ISI below the 25th percentile. Based on this definition, the optimal cutoff value for HOMA-IR determined by ROC analysis was approximately 2.5. Although the optimal cutoff value for HOMA-IR remains controversial [26], several large epidemiological studies have adopted a threshold of 2.5 or higher to indicate clinically significant IR [27, 28]. This concordance supports the validity of the operational definition used in this study. The optimal TyG-BMI cut-off value identified in this cohort was 203.7. We also proposed an estimation formula for the ISI based on TyG-BMI, which may have practical utility in clinical settings where EHC cannot be performed.

In this study, only the anthropometry-adjusted TyG-derived indices maintained significant correlations with ISI regardless of sex, supporting their generalizability and physiological relevance. These findings suggest that anthropometry-adjusted TyG-derived indices, particularly TyG-BMI and TyG-WHtR, may be useful as practical, cost-effective alternatives to clamp-based assessments for large-scale screening or routine clinical practice, particularly when direct measurement of IR is impractical.

Moreover, recent evidence indicates that IR plays a significant role in vascular complications even in individuals with type 1 diabetes [29], a population traditionally considered to have insulin-deficiency as its primary pathophysiological feature. For example, reduced insulin sensitivity assessed by the estimated glucose disposal rate is associated with increased platelet activation and impaired inhibition, indicating a maladaptive thrombotic phenotype in individuals with IR [30]. Therefore, surrogate markers of IR, such as TyG-BMI, may also be valuable in type 1 diabetes, where performing the clamp technique is often challenging.

Recent studies reported that TyG-BMI is associated with multiple metabolic outcomes, including metabolic dysfunction-associated fatty liver disease [18], cardiovascular disease, and mortality [19, 31], with predictive ability superior to that of the conventional TyG index. Furthermore, a recent study of critically ill patients with ischemic stroke demonstrated that, although the TyG index was more strongly associated with short-term mortality, TyG-BMI showed superior predictive performance for long-term outcomes [32]. These findings further underscore the potential clinical value of incorporating anthropometric adjustments into the TyG index to capture broader metabolic risk more effectively. Notably, although TyG-derived indices and the original TyG index share overlapping information, they are not identical. The TyG index primarily reflects dyslipidemia and hyperglycemia, whereas TyG-derived indices also account for the influence of body composition and adiposity. This distinction suggests that using both indices together may provide complementary insights in different clinical contexts.

The median ISI value obtained in this study was comparable to those reported in a previous Japanese cohort [21], where ISI values ranged approximately from 0.07 to 0.14 across subjects with normal glucose tolerance, impaired glucose tolerance, and type 2 diabetes. This consistency supports the validity of the present ISI measurements and indicates that they fall within the expected range for Japanese adults evaluated by the EHC.

In the present study, the Matsuda index, an established OGTT-based measure of insulin sensitivity, demonstrated discriminative performance comparable to that of anthropometry-adjusted TyG-derived indices. However, calculation of the Matsuda index requires a 75-g oral glucose tolerance test with multiple time-point measurements of plasma glucose and insulin, which limits its feasibility in routine clinical practice and large-scale epidemiologic studies. In contrast, TyG-derived indices can be calculated using fasting glucose, triglyceride levels, and simple anthropometric measurements that are routinely available in clinical settings. Importantly, TyG-derived indices can also be applied to populations in whom OGTT-based measures are not feasible, such as individuals with type 1 diabetes or others for whom glucose loading is inappropriate. Thus, even when diagnostic performance is similar, TyG-derived indices may offer meaningful practical advantages over OGTT-based measures in real-world clinical and epidemiologic contexts.

Nevertheless, this study has some limitations. First, TyG-BMI and TyG-WC incorporate anthropometric components by design, and their discriminative ability for IR is therefore partly influenced by adiposity. In the present cohort, BMI alone also demonstrated strong discriminative performance for IR, which is consistent with the established evidence that overall adiposity is a major determinant of peripheral IR assessed by the EHC. By contrast, the purpose of developing anthropometry-adjusted TyG-derived indices is to construct simple composite markers that integrate anthropometric and metabolic information. In this context, TyG-BMI and TyG-WHtR showed superior performance compared with metabolic indices alone, such as HOMA-IR and the original TyG index. Additional analyses indicated that the contribution of anthropometry to TyG-based indices was primarily additive, without evidence of synergistic multiplicative effects. Accordingly, the potential incremental value of these indices over BMI alone warrants cautious interpretation, particularly in relatively young and lean populations. Rather, they may be positioned as practical composite metabolic markers in settings where insulin-based measurements or clamp-based assessments are not feasible. Second, the relatively small sample size reflects the resource-intensive nature of clamp studies limiting statistical power, particularly for cutoff estimation. Therefore, the optimal cutoff values identified in this study should be interpreted as cohort-specific and exploratory. Instead of this, bootstrap-based sensitivity analyses confirmed the robustness of the AUC estimates, supporting the internal validity of the main findings. Third, although inverse correlations between ISI and TyG-derived indices were observed in both men and women, the small number of female participants may limit the reliability of sex-specific analyses; therefore, these subgroup findings should be interpreted as exploratory. Further validation studies in larger and more diverse cohorts are warranted. Fourth, key determinants of IR, including visceral fat area, adipokines (e.g., adiponectin), serum uric acid, and lifestyle factors, were not available in this study. Although AST and ALT were measured, they were not included in the analyses. These omissions limit a comprehensive evaluation of hepatic, visceral, and lifestyle-related IR, and warrant further investigation.

In conclusion, this study is the first to demonstrate that anthropometry-adjusted TyG-derived indices are simple, effective alternative markers for assessing IR, evaluated through direct comparison with the gold-standard EHC. These findings may support its wider clinical and epidemiological application in the future.

## Supplementary Information

Below is the link to the electronic supplementary material.

Supplementary file1 (PDF 295 KB)

## Data Availability

The data from this study are not publicly available. However, the datasets generated and analyzed during the current study are available from the corresponding author upon reasonable request.
