# Supplementary material for: Anthropometry-adjusted TyG indices improve insulin resistance estimation: an exploratory euglycemic–hyperinsulinemic clamp study in Japanese adults without diabetes
Source: Diabetol Int. 2026 Apr 5;17(2):35. doi: 10.1007/s13340-026-00883-9 (PMC13050668; doi:10.1007/s13340-026-00883-9)
Supplement: Supplementary file 1 — Supplementary file1 (PDF 295 KB) [file 13340_2026_883_MOESM1_ESM.pdf]

Supplementary Materials

Supplementary Table 1. Sex-stratified Spearman correlations between metabolic indices and insulin sensitivity index

Supplementary Table 2. Sensitivity analyses of the diagnostic performance of metabolic indices for detecting insulin resistance using alternative ISI thresholds (lowest 20% and 30%)

Supplementary Table 3. Sex-stratified sensitivity analyses of the diagnostic performance of metabolic indices for detecting insulin resistance defined as the lowest quartile of ISI

Supplementary Table 4. Additive and interaction models assessing the incremental contribution of anthropometric measures to TyG-based prediction of insulin resistance

Supplementary Figure 1. Sex-stratified ROC curves of metabolic indices for detecting insulin resistance

Supplementary Table 1. Sex-stratified Spearman correlations between metabolic indices and insulin sensitivity index

Characteristic	Men (n = 45)			Women (n = 16)		
	ρ	Bootstrap 95% CI	*p*-value	ρ	Bootstrap 95% CI	*p*-value
HOMA-IR	−0.355	−0.634 to −0.014	0.017	−0.174	−0.744 to 0.480	0.520
Matsuda index	0.464	0.160 to 0.690	0.001	0.326	−0.345 to 0.799	0.217
TyG index	−0.309	−0.563 to −0.004	0.039	−0.244	−0.742 to 0.373	0.362
TyG-BMI	−0.546	−0.767 to −0.241	<0.001	−0.650	−0.925 to −0.122	0.006
TyG-WC	−0.506	−0.747 to −0.190	<0.001	−0.718	−0.958 to −0.255	0.002
TyG-WHtR	−0.480	−0.715 to −0.166	<0.001	−0.565	−0.887 to −0.018	0.023

Spearman's correlation coefficients (ρ) and *p*-values for the associations between the insulin sensitivity index (ISI) and metabolic indices in men and women. Correlation analyses were conducted using Spearman's rank correlation method.

Abbreviation: HOMA-IR, homeostasis model assessment of insulin resistance; TyG index, triglyceride-glucose index; TyG-BMI, triglyceride-glucose body mass index; TyG-WC, triglyceride-glucose waist circumference; TyG-WHtR, triglyceride-glucose waist-to-height ratio.

Supplementary Table 2. Sensitivity analyses of the diagnostic performance of metabolic indices for detecting insulin resistance using alternative ISI thresholds (lowest 20% and 30%)

	20%		30%	
	AUC (Bootstrap 95% CI)	*p*	AUC (Bootstrap 95% CI)	*p*
HOMA-IR	0.678 (0.453 to 0.878)	0.1122	0.678 (0.516 to 0.827)	0.033
Matsuda index	0.780 (0.582 to 0.941)	0.009	0.759 (0.612 to 0.888)	0.001
TyG index	0.753 (0.590 to 0.896)	0.004	0.697 (0.543 to 0.835)	0.015
TyG-BMI	0.790 (0.607 to 0.942)	0.004	0.821 (0.690 to 0.929)	<0.001
TyG-WC	0.753 (0.590 to 0.896)	0.004	0.763 (0.625 to 0.883)	<0.001
TyG-WHtR	0.798 (0.644 to 0.919)	<0.001	0.807 (0.687 to 0.907)	<0.001

Area under the receiver operating characteristic curve (AUC) with 95% confidence intervals for each metabolic index in detecting insulin resistance, defined using alternative insulin sensitivity index (ISI) thresholds (lowest 20% and lowest 30%). P values indicate the statistical significance of each AUC relative to the null value of 0.5 (bootstrap-based two-sided test).

Abbreviation: AUC, area under the curve; HOMA-IR, homeostasis model assessment of insulin resistance; TyG index, triglyceride-glucose index; TyG-BMI, triglyceride-glucose body mass index; TyG-WC, triglyceride-glucose waist circumference; TyG-WHtR, triglyceride-glucose waist-to-height ratio.

Supplementary Table 3. Sex-stratified sensitivity analyses of the diagnostic performance of metabolic indices for detecting insulin resistance defined as the lowest quartile of ISI

	Men (n = 45)		Women (n = 16)	
	AUC (Bootstrap 95% CI)	*p*	AUC (Bootstrap 95% CI)	*p*
HOMA-IR	0.692 (0.491 to 0.870)	0.062	0.638 (0.000 to 1.000)	0.744
Matsuda index	0.765 (0.580 to 0.950)	0.008	0.813 (0.484 to 1.000)	0.313
TyG index	0.698 (0.571 to 0.917)	0.012	0.614 (0.071 to 1.000)	0.610
TyG-BMI	0.855 (0.698 to 0.973)	<0.001	0.819 (0.536 to 1.000)	0.033
TyG-WC	0.814 (0.654 to 0.942)	<0.001	0.948 (0.795 to 1.000)	0.011
TyG-WHtR	0.808 (0.636 to 0.944)	<0.001	0.948 (0.795 to 1.000)	0.011

Receiver operating characteristic (ROC) analyses were performed separately in men and women, defining insulin resistance as the lowest 25th percentile of the insulin sensitivity index (ISI), consistent with the main analysis. P values indicate the statistical significance of each AUC relative to the null value of 0.5 (bootstrap-based two-sided test).

Abbreviation: AUC, area under the curve; HOMA-IR, homeostasis model assessment of insulin resistance; TyG index, triglyceride-glucose index; TyG-BMI, triglyceride-glucose body mass index; TyG-WC, triglyceride-glucose waist circumference; TyG-WHtR, triglyceride-glucose waist-to-height ratio.

Supplementary Table 4. Additive and interaction models assessing the incremental contribution of anthropometric measures to TyG-based prediction of insulin resistance

Model	N	Apparent AUC	Optimism-corrected AUC	Bootstrap 95% CI	LRT *p* (additive)	LRT *p* (interaction)
**TyG**	61	0.739	0.739	0.739 to 0.739		
BMI-related models						
BMI	61	0.824	0.824	0.824 to 0.824		
TyG+BMI	61	0.836	0.818	0.793 to 0.838	0.0006	
TyG+BMI+ interaction	61	0.836	0.812	0.779 to 0.839		0.175
WC-related models						
WC	61	0.779	0.779	0.779 to 0.779		
TyG+WC	61	0.796	0.776	0.746 to 0.799	0.021	
TyG+WC+ interaction	61	0.800	0.781	0.744 to 0.804		0.588
WHtR-related models						
WHtR	61	0.831	0.830	0.831 to 0.831		
TyG+WHtR	61	0.840	0.824	0.794 to 0.840	0.001	
TyG+WHtR+ interaction	61	0.838	0.809	0.764 to 0.839		0.787

Insulin resistance (IR) was defined as the lowest quartile of the clamp-derived insulin sensitivity index (ISI). Apparent and optimism-corrected areas under the receiver operating characteristic curve (AUCs) are presented. Optimism-corrected AUCs were estimated using 10,000 bootstrap resamples to account for potential overfitting in this relatively small clamp-based cohort, and 95% confidence intervals were derived from the bootstrap distribution of optimism-corrected AUCs.

Additive models included the TyG index and each anthropometric measure (body mass index [BMI], waist circumference [WC], or waist-to-height ratio [WHtR]) as separate covariates. Interaction models additionally included a multiplicative interaction term between the TyG index and the corresponding anthropometric measure (TyG × BMI, TyG × WC, or TyG × WHtR), along with their main effects.

Likelihood ratio tests (LRTs) were performed on the original dataset to compare nested models (TyG vs. TyG + anthropometry for additive models, and TyG + anthropometry vs. interaction models).

Supplementary Figure 1. Sex-stratified ROC curves of metabolic indices for detecting insulin resistance

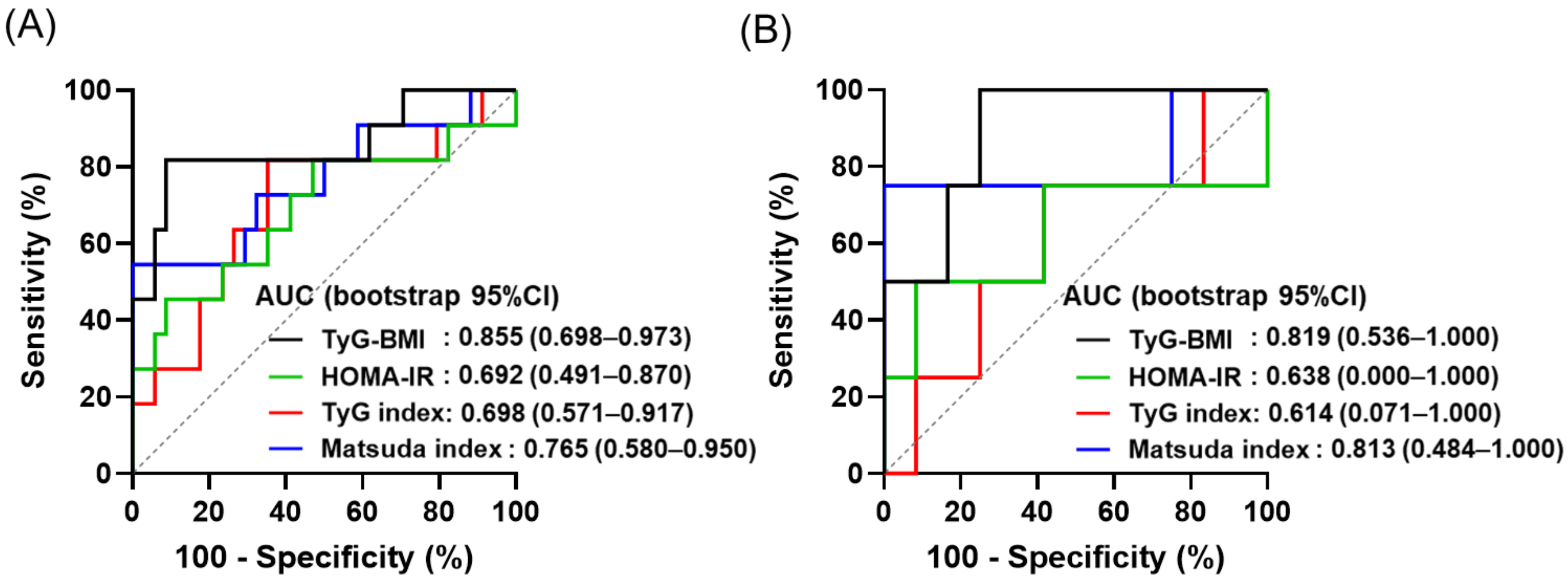

Receiver operating characteristic (ROC) curves of TyG-derived indices for detecting insulin resistance defined as the lowest quartile of the insulin sensitivity index (ISI), stratified by sex: (A) men and (B) women. AUC values with bootstrap 95% confidence intervals are shown.
